# Historical watershed stressors for the Laurentian Great Lakes

**DOI:** 10.1002/gdj3.53

**Published:** 2018-10-29

**Authors:** Euan D. Reavie, Meijun Cai, Terry N. Brown

**Affiliations:** ^1^ Natural Resources Research Institute University of Minnesota Duluth Duluth Minnesota; ^2^ National Health and Environmental Effects Research Laboratory US Environmental Protection Agency Duluth Minnesota

**Keywords:** agriculture, Great Lakes, population, stressors, watershed

## Abstract

This report provides a detailed set of historical stressor data for 60 watersheds comprising the Laurentian Great Lakes basin. Archival records were transcribed from public records to create quantitative data on human activities: population, mining, deforestation, and agriculture. Yearly records of stressors are provided from 1780 through 2010. These data may be used to track historical impacts on Great Lakes coastal and open water conditions. They may further be used to examine corresponding effects on response variables such as biological communities quantified during monitoring and palaeoecological programmes.

**Open Practices:**

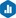

This article has earned an Open Data badge for making publicly available the digitally‐shareable data necessary to reproduce the reported results. The data is available at https://doi.org/10.1594/PANGAEA.885879. Learn more about the Open Practices badges from the Center for Open Science: https://osf.io/tvyxz/wiki.

## 1 INTRODUCTION

Targeting of remedial efforts in lakes requires quantifying anthropogenic stressors that differ spatially and in relative impact. Although historical water quality data can be sparse, human activities that affect water quality, such as mining, agriculture, and shoreline development, more commonly have long‐term archives that have been maintained by government agencies. For decades, researchers have used palaeolimnology to substitute for missing information on historical impacts in aquatic systems (Smol, 2009), including a suite of studies on the Laurentian Great Lakes (Reavie et al., 2017; Schelske & Stoermer, 1971; Schelske, Stoermer, Conley, Robbins, & Glover, 1983; Sgro & Reavie, 2018; Shaw Chraïbi, Kireta, Reavie, Cai, & Brown, 2014; Stoermer, Emmert, Julius, & Schelske, 1996; Stoermer, Kociolek, Schelske, & Conley, 1985a, 1987; Stoermer, Wolin, Schelske, & Conley, 1985b; Stoermer, Wolin, & Schelske, 1993; Wolin, Stoermer, Schelske, & Conley, 1988). While a detailed account of contemporary watershed stressors has been compiled for the Great Lakes (Danz et al., 2007), we further compiled historical stressor data to compare with palaeolimnological records. So far, we have used these data to relate historical anthropogenic activities with fossil diatom trends and geochemistry in Lake Superior (Shaw Chraïbi et al., 2014) and Lake Erie (Sgro & Reavie, 2018). The sediment cores used for comparison were collected from deep, pelagic regions in the lakes, so lake‐wide stressor information (i.e., from all sub‐watersheds around a lake) was relevant, but there is potential to compare nearshore conditions with more localized watershed stressors. For instance, local restoration planning is often focused on maintaining ecosystem services (Bullock, Aronson, Newton, Pywell, & Rey‐Benayas, 2011) where those services are greatest. Furthermore, having a retrospective of stress can enhance strategic targeting of restoration by indicating the most important stressors and whether conditions are improving.

## 2 METHODS

In order to provide stressor data for local watersheds, a set of 5,971 watersheds covering the US and Canadian Great Lakes basin had been delineated using ESRI ArcGIS ArcHydro extensions, as described in Hollenhorst, Brown, Johnson, Ciborowski, and Host (2007) and Host, Brown, Hollenhorst, Johnson, and Ciborowski (2011). For this project, they were agglomerated into 60 watersheds of approximately equal size. Using the St. Lawrence Seaway as a natural break, watersheds from the original 5,971 were collected in shoreline order until an area approximately 1/60th the basin total area was reached. At that point, a GIS union operation was used to merge the small watersheds, and collection for the 60 new “sub‐watersheds” was started. These sub‐watersheds are the primary spatial units of the stressor data.

Spatial and temporal dynamics of land‐use patterns were collected from governmental historical records (Supplement S1; https://doi.org/10.1594/pangaea.885879) and a database was populated with information from various Internet and GIS resources. Land use included agriculture, population, mining, and forestry (Table 1). Transcribed records combined a date (census dates, years of operation for mines), the land use in question, the level of the land use (acres, people, mines), and the spatial distribution of the land use (county boundaries, coordinates for cities, mines, and other phenomena). Historical items were placed on a timeline with annual time‐steps from 1780 through 2010, though this compilation was limited by the accuracy and completeness of the historical archives. Acquiring early data for the whole basin was not possible for some parameters (pre‐1790 population, pre‐1850 agriculture). For years with missing data, we used linear interpolations between adjacent years with data to estimate active stress (e.g., agricultural area) for a given watershed.

**Table 1:** Watershed stressors characterized for this report and their associated units

Stressor variable	Unit
New mines	Number of new mines opened in a given year
Mining stress	Unitless, based on cumulative effects of mines and a decay curve (Section 2.1)
Taconite waste discharge	Unitless, ranging from 0 (none) to 1 (maximum); Lake Superior only
Agriculture	Acres of agricultural area
Population	Number of human residents
Forestry	Acres of forested area

**Figure 1:**
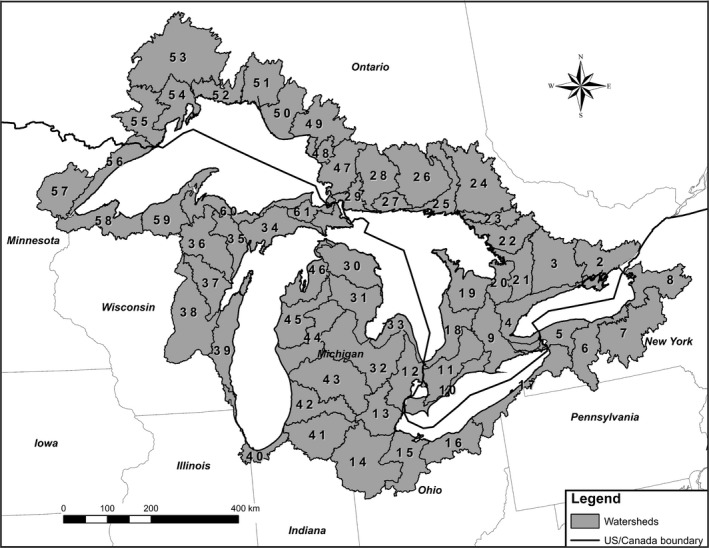
Map of the numbered watersheds that were delineated to characterize regional stress around the Great Lakes

**Figure 2:**
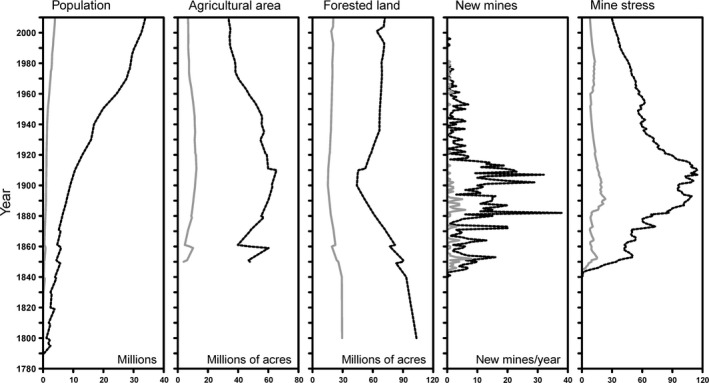
An example of summarized stressor data for all Great Lakes watersheds (2–61) (black lines) and for Lake Huron (watersheds 18–33) (grey lines)

The stressor value associated with each yearly record (stressor value, year) was rendered into a GIS raster grid specific to each land‐use type. The value of each land use was summarized within 60 watersheds covering the basin, numbered 2 through 61 (Figure 1), each watershed capturing a major river and its upgradient catchment. (Sub‐watershed 1 was a St. Lawrence River catchment and was discarded.) Although smaller watersheds would have been possible in some areas, we selected this watershed size based on the overall spatial resolution of historical data. County‐based agricultural land, forested land, and population data were converted to watershed‐based stressors using area‐weighted averaging, while mining data were simply summarized for each watershed based on mine coordinates. Supplement S1 provides references for specific data sources in case a reader was interested in stressor data for more specific locales. We provide the full stressor dataset for all watersheds in Supplement S2 (https://doi.org/10.1594/pangaea.885879). If desired, postprocessing of these data would allow for reporting of land‐use activities for a particular date and/or changes in land‐use for a particular range of years. Historical land‐use data could be generated for all watershed groups combined or for selected subsets of watershed groups (e.g., one lake). Figure 2 provides an example of some compiled data; all stressor data for the Great Lakes basin and data compiled for the watersheds around Lake Huron.

Additional details about the four stressor characters and how they were summarized are provided below.

### 2.1 Mining (USGS, 1844)

Given its importance to the economies of Great Lakes states and provinces, and its well‐known association with water and atmospheric pollution, it was imperative to summarize mining‐related stress. The number of active (and potentially polluting) mines at a given time was difficult to determine from historical records. However, good records were available on the establishment of new mines. The appearance of new mines, in itself, may be a satisfactory tracer of resulting stress in aquatic ecosystems. For instance, the rise of new mining facilities in the vicinity of Sault Ste. Marie was closely associated with cadmium pollution as inferred from a sediment core in Lake George, just upstream from Lake Huron (Reavie et al., 2005). In addition to summarizing new mines (i.e., the number of newly opened mine facilities in a given watershed in a given year), we calculated an additional stressor parameter that roughly estimates the prolonged impacts of mining by applying an arbitrary decay function to the new mine data, forming a “mining stress” indicator. Given the likely high variation in long‐term impacts from mines, we consider this decay constant as highly uncertain, such as attributing the same stress from a cobalt mine and a limestone quarry. While we did not apply such judgements, we wanted to show how cumulative and persistent effects of mining might look. The mining stress level *S* starts at zero and changes per year according to:

$$\frac{dS}{dy}=N_{y}-{\left(0.1\times \left(S_{y-1}+N_{y}\right)\right)}^{2},$$

where *S*_*y*_ is the stress level in year *y* and *N*_*y*_ is the number of new mines in year *y*. This formula generates a unitless stressor value that may be considered the lingering effect of mining activity in a watershed. For instance, the year a mine is established it contributes 0.99 mining stress units to the total for its respective watershed. A year later its contribution is approximately 0.98 units. Etc.

An additional stressor variable characterized taconite waste discharge in Lake Superior at the Reserve Mining facility in Silver Bay (watershed 56). Approximately 500 million tonnes of taconite tailings were discharged to the lake between 1955 and 1980 (Weston, 1971), and the legacy of this contamination appears as geochemical markers in lake sediment cores (Shaw Chraïbi et al., 2014). Quantifying the volume of discharge for a given year was difficult, so we applied an arbitrary scale in which 1.0 represents maximum processing output of the facility.

### 2.2 Agriculture (Canada Census Office 1880–2006, USDA 1850–2002, 2007)

Agriculture largely comprised rural land classification data generally collected as census to determine property values and necessary permits. The Census of Agriculture, taken every 5 years, is a complete count of US farms and ranches. For 156 years (1840–1996), the US Department of Commerce, Bureau of the Census was responsible for collecting data for the Census of Agriculture. In 1997, responsibility transferred to the US Department of Agriculture's National Agricultural Statistics Service (NASS). Agricultural census data for Canada was available approximately every 10 years. In many cases, data were available for farms indicating size and type, inventory, and values for crops and livestock. Unfortunately, these details were historically erratic, so we focused on documented agricultural area as the tracer for stress.

### 2.3 Population (Canada Dominion Bureau of Statistics, 1871–2011, US Department of Commerce, USBC, 1850–2010

Census data for rural and urban populations were fairly consistently available over the last two centuries. While compiling these data was time consuming, little extrapolation was required to fill data gaps.

### 2.4 Forests (Supplement S1, Reavie, Cai, & Brown, 2018)

Technically these data do not represent a “stressor” in the same way as the other parameters, but a long‐term decline in forested area (deforestation) reflects watershed stress. It is well‐known that deforestation results in increased runoff and, typically in association with croplands, increases nutrient supplies to aquatic systems (Hasler, 1947). These data were available from forest inventory records that characterized forest land area for each county in the United States. Periodic inventories of forested land were used to determine forest extent and removals. Without a forest inventory survey in Canada, forested area in Canada was calculated as: Total land area minus agricultural, water, and urban areas.

Urban area data were provided by the US Department of Commerce and the US Census Bureau 1850–2010) and the Canada Dominion Bureau of Statistics 1871–2011.

It is our hope that these data and source summaries are useful, and we welcome discussions with interested researchers on ways to further refine or process the dataset. While maintenance is not currently funded, this land‐use dataset will likely be developed and refined in the future, particularly with the addition of post‐2010 data. Nonetheless, at this time, the compiled results provide a clear picture of historical activities around the Great Lakes.
